# Simulating grazing beef and sheep systems

**DOI:** 10.1016/j.agsy.2021.103307

**Published:** 2022-01

**Authors:** L. Wu, P. Harris, T.H. Misselbrook, M.R.F. Lee

**Affiliations:** a Sustainable Agriculture Sciences, Rothamsted Research, North Wyke, Devon EX20 2SB, UK; b Bristol Veterinary School, University of Bristol, Langford, Somerset BS40 5DU, UK

**Keywords:** SPACSYS, North Wyke Farm Platform, Grazing, Modelling, Liveweight

## Abstract

**CONTEXT:**

Ruminant livestock make an important contribution to global food security by converting feed that is unsuitable for human consumption into high value food protein, demand for which is currently increasing at an unprecedented rate because of increasing global population and income levels. Factors affecting production efficiency, product quality, and consumer acceptability, such as animal fertility, health and welfare, will ultimately define the sustainability of ruminant production systems. These more complex systems can be developed and analysed by using models that can predict system responses to environment and management.

**OBJECTIVE:**

We present a framework that dynamically models, using a process-based and mechanistic approach, animal and grass growth, nutrient cycling and water redistribution in a soil profile taking into account the effects of animal genotype, climate, feed quality and quantity on livestock production, greenhouse gas emissions, water use and quality, and nutrient cycling in a grazing system.

**METHODS:**

A component to estimate ruminant animal growth was developed and integrated with the existing components of the SPACSYS model. Intake of herbage and/or concentrates and partitioning of the energy and protein contained in consumed herbage and/or concentrates were simulated in the component. Simulated animal growth was validated using liveweight data from over 200 finishing beef cattle and 900 lambs collected from the North Wyke Farm Platform (NWFP) in southwest England, UK, between 2011 and 2018. Annual nitrous oxide (N_2_O), ammonia, methane and carbon dioxide emissions from individual fields were simulated based on previous validated parameters.

**RESULTS AND CONCLUSIONS:**

A series of statistical indicators demonstrated that the model could simulate liveweight gain of beef cattle and lamb. Simulated nitrogen (N) cycling estimated N input of 190 to 260 kg ha^−1^, of which 37–61% was removed from the fields either as silage or animal intake, 15–26% was lost through surface runoff or lateral drainage and 1.14% was emitted to the atmosphere as N_2_O. About 13% of the manure N applied to the NWFP and excreta N deposited at grazing was lost via ammonia volatilisation.

**SIGNIFICANCE:**

The extended model has the potential to investigate the responses of the system on and consequences of a range of agronomic management and grazing strategies. However, modelling of multi-species swards needs to be validated including the dynamics of individual species in the swards, preferential selection by grazing animals and the impact on animal growth and nutrient flows.

## Highlights

• Animal-plants-environment interactions in complexed livestock systems need process-based models to be investigated.
• Functions in an existing model were extend and validated based on a data-rich monitoring farm platform.
• The extended model accurately simulated liveweight changes of beef and lamb within the natural variations expected.
• Simulated emissions (N_2_O, NH_3_, CH_4_ and CO_2_) and components in the nitrogen budget are compatible with the published values.
• The model has the potential to investigate the consequences of agronomic practices and grazing strategies.

## Graphical abstract

**Figure:**
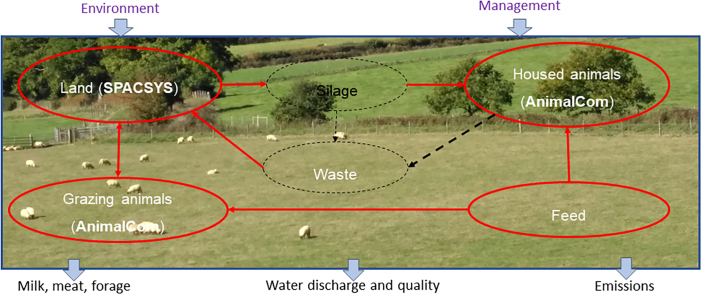

## 1 Introduction

We are at a critical juncture for global livestock production as competing requirements for maximal productivity and minimal pollution have driven the requirement for sustainable intensification (Springmann et al., 2018). Ruminants make an important contribution to global food security by converting feed that is unsuitable for human consumption into high value food protein, demand for which is currently increasing at an unprecedented rate because of increasing global population and income levels (Tilman and Clark, 2014). Reduction in red meat and dairy intake is increasingly seen as a pathway to improving human and environmental health (e.g. Westhoek et al., 2014), but globally, ruminant livestock will be important for the foreseeable future and demonstrating methods for sustainable production will become increasingly important. Sustainable intensification of ruminant livestock may be applied to pastoral grazing, mixed-cropping, feedlot, and housed production systems. All these systems have associated environmental impacts such as water and air pollution where greenhouse gas (GHG) emissions, soil degradation and erosion are all of particular concern. In addition, factors affecting production efficiency, product quality, and consumer acceptability, such as reduced animal fertility, health and welfare, also impact on the sustainability of ruminant production systems. These challenges necessitate multidisciplinary solutions that can only be properly researched, implemented and tested in real-world production systems (Eisler et al., 2014). As a consequence, there is a call to ‘redesign’ livestock systems, including the integration of both crops and livestock (Dumont et al., 2014). These more complex systems can be developed and analysed by using models that can predict system responses to environment and management.

Several reviews of grassland-based ruminant production models have been published (Bateki et al., 2019; Bryant and Snow, 2008; Snow et al., 2014). In order to simulate ruminant livestock systems, the components of animal genetics (breed), nutrition (forage), management practices and their subsequent impact on the surrounding environment (emissions to air and water) must be considered as a whole in computational models. Several mechanistic process-based simulation models have attempted to simulate the whole system, e.g. the Hurley Pasture Model (Thornley, 1998) and its subsequent revisions - PaSim (Graux et al., 2011), WFM (Neil et al., 1999), GRAZPLAN (Donnelly et al., 2002), GrazeIn (Faverdin et al., 2011), SEDIVER (Martin et al., 2011), e-Dairy (Baudracco et al., 2013) and LiGAPS-Beef (van der Linden et al., 2019). Challenges remain in modelling ruminant systems, due to the symbiotic relationship between rumen microbial anaerobic fermentation and subsequent mammalian metabolism of a combination of derived rumen microbial biomass (microbial protein), fermentation by-products (volatile fatty acids and ammonia) and dietary components which by-pass rumen fermentation. As well as associated microbial activity which influences lipid profiles (biohydrogenation), atmospheric pollutants (methanogenesis) and which ultimately drives the partitioning and retention (milk, live weight, faeces, urine) of dietary nutrients. A systems approach to investigate ruminant production through modelling and simulation is therefore recommended (Dougherty et al., 2019; Hirooka, 2010).

The SPACSYS model (Wu et al., 2007) is a weather-driven dynamic simulation model at a field scale with up to a daily step. Since it was first published in 2007, it has been developed to provide added functionality, e.g. the impact of vernalisation on overwinter crops (Bingham and Wu, 2011), biological nitrogen (N) fixation by legumes (Liu et al., 2013), microbial-based N_2_O emissions (Wu et al., 2015) and soil phosphorus (P) cycling (Wu et al., 2019). The model can simulate the interactions of soil carbon (C), N and P, plant growth and development, water re-distribution and heat transformation in agricultural fields. The model has been applied to grassland systems in the assessment of GHG emissions (Abalos et al., 2016), responses to environmental change (Ehrhardt et al., 2018; Wu et al., 2016) under various climatic and soil conditions, nutrient cycling (Carswell et al., 2019b) and C fluxes (Sándor et al., 2020). However, as there is no component to describe animal growth, simulations involving animals have required pre-processing and direct input of data on grass intake rate and nutrient returns in animals, rather than deriving directly from animal performance, constraining model application.

This study presents a framework that dynamically models animal and grass growth, nutrient cycling and water redistribution in a soil profile taking into account the effects of animal genotype, climate, feed quality and quantity on livestock production, GHG emissions, water use and quality, and nutrient cycling in a grazing system, using a process-based and mechanistic modelling approach. Simulated animal growth was validated using liveweight data collected from over 200 finishing beef cattle and 900 lambs collected from the North Wyke Farm Platform (NWFP) in southwest England, UK, between 2011 and 2018. The framework could potentially integrate economic, environmental and social factors to provide decision makers with the ability to forecast, interpret and respond to potential threats to UK livestock production systems.

## 2 Materials and methods

### 2.1 SPACSYS model

**Fig. 1:**
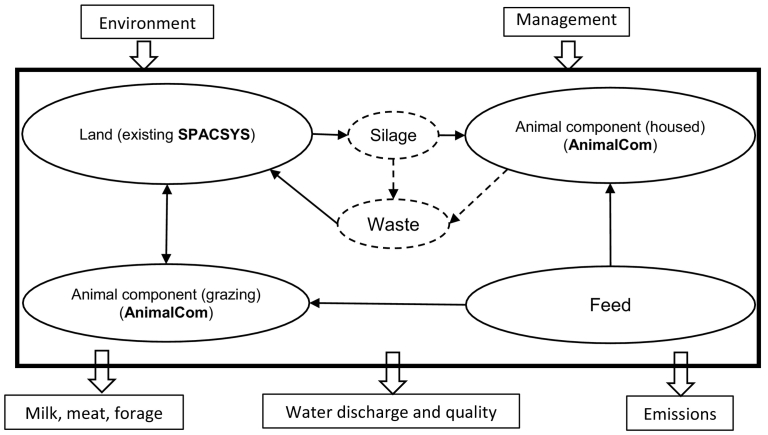
Extension of the SPACSYS model, component linkages, inputs and outputs. Solid lines show the components and linkages included in the latest version of the model. Dashed lines indicate components and flows for future inclusion.

In this study, a component to estimate ruminant animal growth, AnimalCom, was developed, implemented and integrated with the existing components of SPACSYS (Fig. 1). Existing model components are published in detail elsewhere (Wu et al., 2007, Wu et al., 2015, Wu et al., 2019), while the new AnimalCom is described here.

### 2.2 AnimalCom

**Fig. 2:**
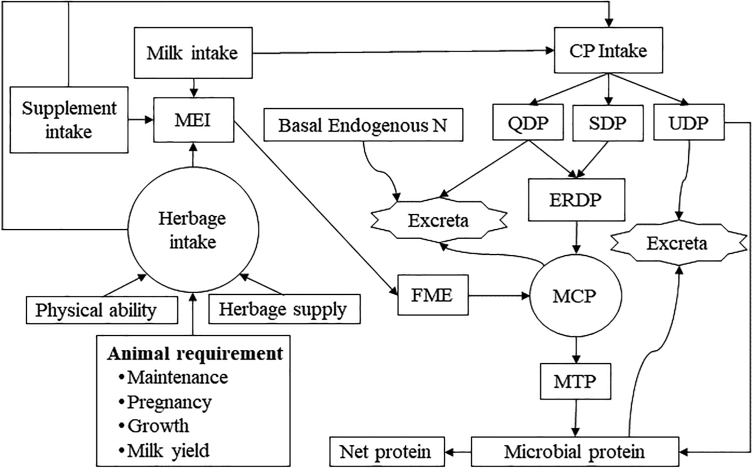
A schematic representation of the factors limiting intake and the metabolisable energy and protein system. MEI: metabolisable energy intake (MJ head^−1^ day^−1^); FME: fermentable metabolisable energy (MJ head^−1^ day^−1^); CP: crude protein (g head^−1^ day^−1^); QDP: quickly degradable protein content (g head^−1^ day^−1^); SDP: slowly degradable protein content (g head^−1^ day^−1^); UDP: undegradable dietary protein content (g head^−1^ day^−1^); ERDP: effective rumen degradable protein content (g head^−1^ day^−1^); MCP: microbial crude protein supply (g head^−1^ day^−1^); MTP: microbial true protein (g head^−1^ day^−1^); and MTP: true protein content of MCP (g head^−1^ day^−1^).

The AnimalCom component consists of two parts: intake of herbage and/or concentrates and partitioning of the energy and protein contained in consumed herbage and/or concentrates. Herbage intake by grazing ruminant livestock is assumed to be regulated by one of three factors (Loewer et al., 1983): a) the physiological limit on intake (or thermodynamic limit), b) the feed availability and c) the physical ability of the animal to consume feed (Fig. 2). The first factor is partially determined by the energy requirement of the animal. There are several systems developed for nutritional evaluation (Tedeschi et al., 2005), where the description below is mainly based on the UK Agricultural and Food Research Council metabolizable energy (ME) and protein (MP) system (Agricultural and Food Research Council, 1993) in which the dynamics of the rumen microbial population plays a vital role.

Metabolisable energy intake (MEI) through grazing and concentrate feeds is partitioned among that required for maintenance, pregnancy (for cow and dry ewe only), growth and fattening, and milk production (for cow and lactating ewe only). In general, the requirement for animal maintenance is given the highest priority, then pregnancy, and the lowest for milk production and liveweight gain.

#### 2.2.1 Energy requirements

Physiological ME requirement (MJ head^−1^ day^−1^), as shown in Fig. 2 as “animal requirement” is defined by a generic term:

(1) $$E_{\mathrm{PH}\_C}=E_{\mathrm{req}-\mathrm{main}}+E_{\mathrm{req}-\mathrm{preg}}+E_{\mathrm{req}-\mathrm{growth}}+E_{\mathrm{req}-\mathrm{milk}}\;$$

where *E*_*req-main*_ is the ME requirement for maintenance (MJ head^−1^ day^−1^), *E*_*req-growth*_ is the ME requirement for growth and fattening (MJ head^−1^ day^−1^), *E*_*reg-preg*_ is the ME requirement for pregnancy (MJ head^−1^ day^−1^), and *E*_*req-milk*_ is the ME requirement for milk production (MJ head^−1^ day^−1^).

##### 2.2.1.1 Energy requirement for maintenance

Following Agricultural and Food Research Council (1993), *E*_*req-main*_ including fasting metabolism and activity allowance for the animal is estimated by:

(2) $$E_{\mathrm{req}-\mathrm{main}}=\frac{\left[a\;{\left(\frac{\mathrm{LWT}}{1.08}\right)}^{b}\right]+c∙\mathrm{LWT}}{e_{e-\mathrm{main}}}$$

where *LWT* is the live weight of the animal (kg), *a*, *b* and *c* are empirical parameters and *e*_*e-main*_ is the efficiency of utilisation of ME for maintenance.

For sheep, it has been documented for some time that the AFRC (1993) model may underestimate maintenance energy requirement (e.g. Yang et al., 2019). The equation adopted here is therefore that used in the UK inventory model for agricultural GHG emissions, developed by Steven Anthony (pers comm. ADAS, 2021). Consequently, the requirement is estimated as:

(3) $$E_{\mathrm{req}-\mathrm{main}}=\frac{k_{2}k_{4}{\left(\mathrm{LWT}-0.0097L{\mathrm{WT}}^{1.2735}\right)}^{0.75}∙e^{-k_{3}∙\min\left(6,\frac{A_{\mathrm{sheep}}}{365}\right)}∙\left[1+0.26∙\frac{\max\left(0,D_{w}-A_{\mathrm{lamb}}\right)}{D_{w}}\right]}{e_{e-\mathrm{main}}}$$

where *k*_*2*_, *k*_*3*_ and *k*_*4*_ are constants and setting 0.26, 0.03, and 1.0 (female and castrate male) or 1.15 (entire male), respectively; *D*_*w*_ is the weaning length of sheep (days); *A*_*sheep*_ *and A*_*lamb*_ are the age (days) of sheep and the lamb, respectively; min and max are math functions for a minimum and maximum value of two values, respectively. This adaptation to AFRC (1993) added a further 9% of MEI to the requirement.

##### 2.2.1.2 Energy requirement for pregnancy

*E*_*reg-preg*_ is estimated as (Agricultural and Food Research Council, 1993):

(4) $$E_{\mathrm{req}-\mathrm{preg}}=\frac{10^{\left(a_{e}-b_{e}\cdot e^{-c_{e}∙D_{\mathrm{preg}}}\right)}\cdot d_{e}\cdot e^{c_{e}∙D_{\mathrm{preg}}}}{e_{e-\mathrm{preg}}}$$

where *a*_*e*_, *b*_*e*_, *c*_*e*_ and *d*_*e*_ are parameters, *D*_*preg*_ is the pregnancy period (days) and *e*_*e-preg*_ is the efficiency of utilisation of ME for the conceptus.

##### 2.2.1.3 Energy requirement for liveweight change

*E*_*req-growth*_ is based on the potential live weight growth rate that is expressed as the Gompertz function (Lewis et al., 2002; Taylor, 1968):

(5) $$E_{\mathrm{req}-\mathrm{growth}}=\frac{∆W∙e_{g}}{e_{e-\mathrm{growth}}}$$

(6) $$∆W=\frac{1}{g_{f}∙{\mathrm{LWT}}_{m}^{0.3}}\cdot \mathrm{LWT}\cdot \ln\left(\frac{{\mathrm{LWT}}_{m}}{\mathrm{LWT}}\right)$$

where *g*_*f*_ is a Gompertz constant that tends to be smaller as the mature size becomes larger (Emmans and Kyriazakis, 2000), *LWT*_*m*_ is the average weight of the animal at maturity (kg), *e*_*g*_ is the ME requirement per unit live weight increase of the animal (MJ kg^−1^), and *e*_*e-growth*_ is the efficiency of utilisation of ME for liveweight change.

For finishing beef cattle, however, the potential energy requirement for growth and fattening is determined by the potential gain in protein (*ΔP*) and fat content (*ΔF*) of the empty body weight (Topp, 1999):

(7) $$∆W=∆P∙P_{e}+∆F∙F_{e}$$

where *P*_*e*_ (MJ kg^−1^) and *F*_*e*_ (MJ kg^−1^) are the energy values of protein and fat, respectively.

##### 2.2.1.4 Energy requirement for milk production

Energy requirement for milk production from a lactating animal is estimated by:

(8) $$E_{\mathrm{req}-\mathrm{milk}}=\frac{Y_{\mathrm{milk}}∙e_{m}}{e_{e-\mathrm{milk}}}$$

where *e*_*m*_ is the energy requirement per unit milk produced (MJ kg^−1^), *e*_*e-milk*_ is the use efficiency of ME for milk production and *Y*_*milk*_ is potential milk yield (kg head^−1^ d^−1^), that is controlled by a lactation curve described by Wood (1980) and then corrected by the period of milk production and the weeks of calving (and lambing) from the beginning of a year (Mainland, 1985).

(9) $$Y_{\mathrm{milk}}=Y_{\mathrm{init}}∙{\left(\frac{D_{w}}{7}\right)}^{a_{w}}\cdot e^{-b_{w}\cdot \frac{D_{w}}{7}}\cdot \left(1+f_{w}\right)\cdot \left(1+f_{c}\right)$$

where *f*_*w*_ and *f*_*c*_ are parameters to reflect seasonal and calving (lambing) date effects on milk production, which are the tabulated functions of weeks from the beginning of a year. *Y*_*init*_ is the initial yield (kg head^−1^ d^−1^) of milk and affected by lactation number. *a*_*w*_ and *b*_*w*_ are parameters.

The efficiencies of ME utilisation are determined by a linear function of the metabolisability (*M*_*e*_) of gross energy at maintenance. For grazed grasses, it is estimated by:

(10) $$M_{e}=\frac{{\mathrm{ME}}_{g}}{{\mathrm{GE}}_{g}}$$

where *ME*_*g*_ and *GE*_*g*_ are the ME and the gross energy content of the forage (MJ kg^−1^), respectively.

(11) $${\mathrm{ME}}_{g}=\frac{d_{g}∙C_{d-M}}{1000}$$

where *C*_*d-M*_ is the conversion coefficient from digestible to metabolisable energy, with a default value of 16 MJ kg^−1^ (Agricultural and Food Research Council, 1993), and *d*_*g*_ is digestibility of forage, i.e. D-value (g kg^−1^ DM), that is estimated in the model.

*GE*_*g*_ was calculated as (Murray, 1991):

(12) $${\mathrm{GE}}_{g}=0.0065\mathrm{CP}+17.7$$

where *CP* is the crude protein content (g kg^−1^DM) of the grass and estimated by the N content of the grass multiplied by 6.25.

#### 2.2.2 Protein requirements

Following Hulme et al. (1986), the protein requirement for maintenance (*P*_*reg-main*_) is estimated as:

(13) $$P_{\mathrm{req}-\mathrm{main}}=\frac{\left(0.35+0.018\right)∙6.25{\mathrm{LWT}}^{0.75}}{e_{p-\mathrm{main}}}$$

where *e*_*p-main*_ is the conversion efficiency of metabolizable protein to net protein.

The protein requirement for pregnancy (*P*_*reg-preg*_) is estimated as (Agricultural and Food Research Council, 1993):

(14) $$P_{\mathrm{req}-\mathrm{preg}}=\frac{10^{\left(a_{p}-b_{p}\cdot e^{-c_{p}∙D_{\mathrm{preg}}}\right)}\cdot d_{p}\cdot e^{c_{p}∙D_{\mathrm{preg}}}}{e_{p-\mathrm{preg}}}$$

where *a*_*p*_, *b*_*p*_, *c*_*p*_ and *d*_*p*_ are parameters and *e*_*p-preg*_ is the efficiency of utilisation of protein for the conceptus.

Protein requirement for growth is estimated as:

(15) $$P_{\mathrm{req}-\mathrm{growth}}=\frac{138.0\mathrm{\Delta W}}{f_{p-\mathrm{growth}}}$$

where *f*_*p-growth*_ is the fraction of protein in faeces.

The protein requirement for milk production (*P*_*reg-milk*_) is estimated by:

(16) $$P_{\mathrm{req}-\mathrm{milk}}=\frac{P_{\mathrm{per}}\cdot Y_{\mathrm{milk}}\cdot f_{\mathrm{true}}}{e_{p-\mathrm{milk}}}$$

where *P*_*per*_ is the protein percentage in milk, *f*_*true*_ is the fraction of true protein in milk and *e*_*p-milk*_ is the efficiency of utilisation of protein for milk production.

#### 2.2.3 Herbage intake

Mechanisms for the long-term regulation on feed intake are still unclear and will differ between grazing and stall feeding. It was assumed that actual daily intake for the animal (*DMI*, kg DM head^−1^ d^−1^) is determined by the most limiting factor among feed availability, physical ability and physiological requirement for intake:

(17) $$\mathrm{DMI}=\min\left({\mathrm{DMI}}_{G},{\mathrm{DMI}}_{\mathrm{PHYSICAL}},{\mathrm{DMI}}_{\mathrm{PH}}\right)$$

where *DMI*_*PHYSICAL*_ is the physical ability on herbage intake (kg DM head^−1^ d^−1^), *DMI*_*PH*_ is the herbage intake (kg DM head^−1^ d^−1^) based on energy requirements, and *DMI*_*G*_ is the intake rate (kg DM head^−1^ d^−1^) based on herbage availability in the field.

##### 2.2.3.1 Physical ability

Feed intake by the animal is controlled by the rate of passage through and the amount of undigested material in the digestive tract. For cattle, this is used (Kahn and Spedding, 1984):

(18) $${\mathrm{DMI}}_{\mathrm{PHYSICAL}}=\frac{F_{a}∙\mathrm{LWT}}{1-\frac{{\mathrm{dg}}_{\mathrm{DM}}}{1000}\;}$$

and for sheep (Blaxter et al., 1961):

(19) $${\mathrm{DMI}}_{\mathrm{PHYSICAL}}=\frac{F_{a}∙{\mathrm{LWT}}^{0.734}}{1-\frac{{\mathrm{dg}}_{\mathrm{DM}}}{1000}}$$

where *d*_*max*_ and *dg*_*DM*_ are the average faecal DM output rate per unit liveweight (kg DM day^−1^) and digestibility of feed, i.e. D-value (g kg^−1^ DM), respectively.

##### 2.2.3.2 Physiological requirement

DMI_PH_ is regulated by the daily ME requirement of the animal (Topp, 2001), and given by:

(20) $${\mathrm{DMI}}_{\mathrm{PH}}=\frac{E_{\mathrm{PH}\_C}-E_{\mathrm{conc}}}{M_{\mathrm{Fod}}}$$

where *E*_*conc*_ is the daily ME intake rate of concentrates if supplied (MJ head^−1^ day^−1^), and *M*_*Fod*_ is the ME (MJ kg^−1^DM) of ingested herbage, defined by Moran (2005):

(21) $$M_{\mathrm{Fod}}=0.017\times {\mathrm{dg}}_{\mathrm{DM}}-2.0$$

##### 2.2.3.3 Feed availability

When the quantity of herbage available for consumption is less than that required for 95% of maximum daily intake, the daily allowance of green herbage regulates intake. The green herbage allowance is taken to be the green herbage mass above the minimum herbage mass required for grazing. *DMI*_*G*_ was estimated as (Zemmelink, 1980):

(22) $${\mathrm{DMI}}_{G}=I_{\max}{\left[1-e^{-{\left(\frac{H}{I_{\max}}\right)}^{p_{\mathrm{shape}}}}\right]}^{\frac{1}{p_{\mathrm{shape}}}}$$

where *p*_*shape*_ is a constant, *H* is the daily allowance of green herbage for the animal (kg DM head^−1^ d^−1^) and *I*_*max*_ is the maximum daily intake of herbage (kg DM head^−1^ d^−1^) and is described by:

(23) $$I_{\max}=F_{\max}\times {\mathrm{LWT}}^{0.75}$$

where *F*_*max*_ is the maximum DM intake rate per kg of metabolic weight (kg DM (liveweight)^-0.75^ head^−1^ d^−1^).

#### 2.2.4 ME intake partitioning

There are four possible scenarios to partition ME intake depending on ME supply and animal requirements (Tess and Kolstad, 2000; Topp, 1999), meeting: 1) the physiological requirements of the animal (MEI ≥ *E*_*PH_C*_); 2) the maintenance and pregnancy requirements but not the potential energy requirements for milk production and growth and fattening (*E*_*PH_C*_ > MEI ≥ *E*_*req-main*_ + *E*_*req-preg*_); 3) the maintenance requirements but not pregnancy and the potential energy requirements for milk production and growth and fattening are not fulfilled (*E*_*req-main*_ + *E*_*req-preg*_ > MEI ≥ *E*_*req-main*_); and 4) no requirement (MEI < *E*_*req-main*_).

##### 2.2.4.1 Scenario 1

In this case, all the requirements can be met, and potential milk production (Eq. (9)) and growth (Eqs. (6), (7)) will be achieved.

##### 2.2.4.2 Scenario 2

The energy requirements of the animal for maintenance and pregnancy are met. The energy deficit (*ME*_*d*_, MJ head^−1^ d^−1^) for milk and liveweight change is:

(24) $${\mathrm{ME}}_{d}=E_{\mathrm{PH}\_C}-\mathrm{MEI}$$

It was assumed that the energy deficit is partitioned in equal amounts to reductions in potential energy requirements for milk and growth, i.e.

(25) $$\;E_{a\_\mathrm{growth}}=E_{\mathrm{req}-\mathrm{growth}}-\frac{{\mathrm{ME}}_{d}}{2}\;\text{and}\;E_{a\_\mathrm{milk}}=E_{\mathrm{req}-\mathrm{milk}}-\frac{{\mathrm{ME}}_{d}}{2}$$

If *E*_*a*_*growth*_ ≥ 0, milk production and growth are calculated based on *E*_*a*_*growth*_ and *E*_*a*_*milk*_.

If *E*_*a*_*growth*_ < 0, then maternal body tissue will be catabolised for milk production (*ΔE*_*m*_) by:

(26) $$∆E_{m}=\frac{\frac{{\mathrm{ME}}_{d}}{2}-E_{\mathrm{req}-\mathrm{growth}}}{2}$$

with the rate of change in body weight as:

(27) $$∆W=-\frac{∆E_{m}}{N_{l}}$$

and milk production estimated as:

(28) $$Y_{\mathrm{milk}}=e_{e-\mathrm{milk}}\frac{{\mathrm{ME}}_{d}}{2}+k_{\mathrm{bm}}∆E_{m}$$

where *N*_*l*_ is the net energy produced per unit of catabolised liveweight (MJ kg^−1^) and *k*_*bm*_ is the efficiency of utilisation of maternal body tissue for milk production.

##### 2.2.4.3 Scenario 3

The ME requirement for pregnancy (*ΔE*_*p*_) and milk production (*ΔE*_*m*_) are met from maternal tissue catabolism:

(29) $${\mathrm{\Delta E}}_{p}=\left(E_{\mathrm{req}-\mathrm{main}}+E_{\mathrm{req}-\mathrm{preg}}-\mathrm{MEI}\right)\frac{e_{e-\mathrm{preg}}}{k_{\mathrm{bc}}}$$

and

(30) $${\mathrm{\Delta E}}_{m}=\max\left(0,\frac{E_{\mathrm{req}-\mathrm{milk}}-E_{\mathrm{req}-\mathrm{growth}}-{\mathrm{\Delta E}}_{p}}{2}\right)$$

where *k*_*bc*_ is utilisation efficiency of maternal body tissue for pregnancy.

Actual milk production and liveweight change rate are:

(31) $$Y_{\mathrm{milk}}=k_{\mathrm{bm}}∆E_{m}$$

(32) $$∆W=-\frac{∆E_{m}+{\mathrm{\Delta E}}_{p}}{N_{l}}$$

##### 2.2.4.4 Scenario 4

The ME required from maternal body tissue to meet the maintenance (*ΔE*_*ma*_), pregnancy (if needed) and milk production that is estimated by Eq. (31).

(33) $${\mathrm{\Delta E}}_{\mathrm{ma}}=\left(E_{\mathrm{req}-\mathrm{main}}-\mathrm{MEI}\right)$$

(34) $${\mathrm{\Delta E}}_{p}=E_{\mathrm{req}-\mathrm{preg}}\frac{e_{e-\mathrm{preg}}}{k_{\mathrm{bc}}}$$

(35) $${\mathrm{\Delta E}}_{m}=\max\left(0,\frac{E_{\mathrm{req}-\mathrm{milk}}-E_{\mathrm{req}-\mathrm{growth}}-{\mathrm{\Delta E}}_{p}-{\mathrm{\Delta E}}_{\mathrm{ma}}}{2}\right)$$

(36) $$∆W=-\frac{{\mathrm{\Delta E}}_{\mathrm{ma}}+∆E_{m}+{\mathrm{\Delta E}}_{p}}{N_{l}}$$

#### 2.2.5 Protein degradation

Protein degradation in the rumen and efficiency values for the degradation followed the metabolisable protein system proposed by AFRC (1993). During the degradation of intake crude protein, fermentable metabolisable energy from *MEI* is incorporated to estimate microbial crude protein supply (MCP), shown in Fig. 2.

#### 2.2.6 GHG emissions

##### 2.2.6.1 CO_2_ emissions

Following Kirchgessner et al. (1991), CO_2_ emission rate (g C head^−1^ d^−1^) from an adult dairy or beef cow was estimated by:

(37) $$E_{\mathrm{CO}2}=\left(-1.4+0.43\mathrm{DMI}-0.045{\mathrm{LWT}}^{0.75}\right)∙1000∙\frac{12}{44}$$

However, for a lamb or ewe, the rate was estimated (CIGR, 2002; Haque et al., 2014) by:

(38) $$E_{\mathrm{CO}2}=\frac{180\times 24\mathrm{HP}}{1000+4\left(20-T_{a}\right)}∙\frac{12P_{a}}{8.31\left(273.17+T_{a}\right)}∙\frac{1}{1000}$$

where *P*_*a*_ is atmospheric pressure (Pa), *T*_*a*_ is air temperature (°C) and *HP* is heat production (Watt):

(39) $$\mathrm{HP}=\left\{\begin{array}{l} 6.4{\mathrm{LWT}}^{0.75}+145∆W\;\left(\text{for lamb or non}-\text{lactating}\;\mathrm{ewe}\right)\\ 6.4{\mathrm{LWT}}^{0.75}+33Y_{\mathrm{milk}}\;\left(\text{for lactating}\;\mathrm{ewe}\right) \end{array}\right)$$

##### 2.2.6.2 Methane (CH_4_) emissions

For dairy and beef cattle, the regression equation from Yan et al. (2009) was used to estimate the enteric CH_4_ emission rate (g CH_4_ head^−1^d^−1^):

(40) $$E_{\mathrm{CH}4}=\left[\left(1.749-\frac{12.18\mathrm{ME}}{\mathrm{GE}}+\frac{10.74\mathrm{DE}}{\mathrm{GE}}\right)∙\mathrm{GE}∙\mathrm{DMI}-14.0\right]∙\frac{16P_{a}}{8.31\left(273.17+T_{a}\right)}$$

where *GE, ME and DE* (MJ kg^−1^DM) are the gross energy, ME and digestible energy in the dry matter intake, including forage and concentrate, respectively, and *GEI* is the gross energy intake (MJ head^−1^d^−1^). Values for *GE*, *ME* and *DE* were estimated as the weighted average across forage and concentrate.

Following Stergiadis et al. (2015), digestible energy from grass was estimated as:

(41) $${\mathrm{DE}}_{g}=-10.2+\frac{45.1\mathrm{CP}}{6.25\times 1000}+1.29{\mathrm{GE}}_{g}$$

For sheep and lamb, the equation proposed by Blaxter and Clapperton (1965) was adopted:

(42) $$E_{\mathrm{CH}4}=\left[1.3+\frac{11.2\mathrm{DE}}{\mathrm{GE}}-\frac{E_{\mathrm{PH}\_C}}{E_{\mathrm{req}-\mathrm{main}}}∙\left(2.37-\frac{5\mathrm{DE}}{\mathrm{GE}}\right)\right]∙\frac{1}{100}∙\mathrm{GE}∙\mathrm{DMI}∙\frac{1}{0.05565}∙\frac{16P_{a}}{8.31\left(273.17+T_{a}\right)}$$

where 0.05565 (MJ g^−1^) is the energy generated by CH_4_ (de Haas et al., 2011).

### 2.3 Case study grazing system

**Fig. 3:**
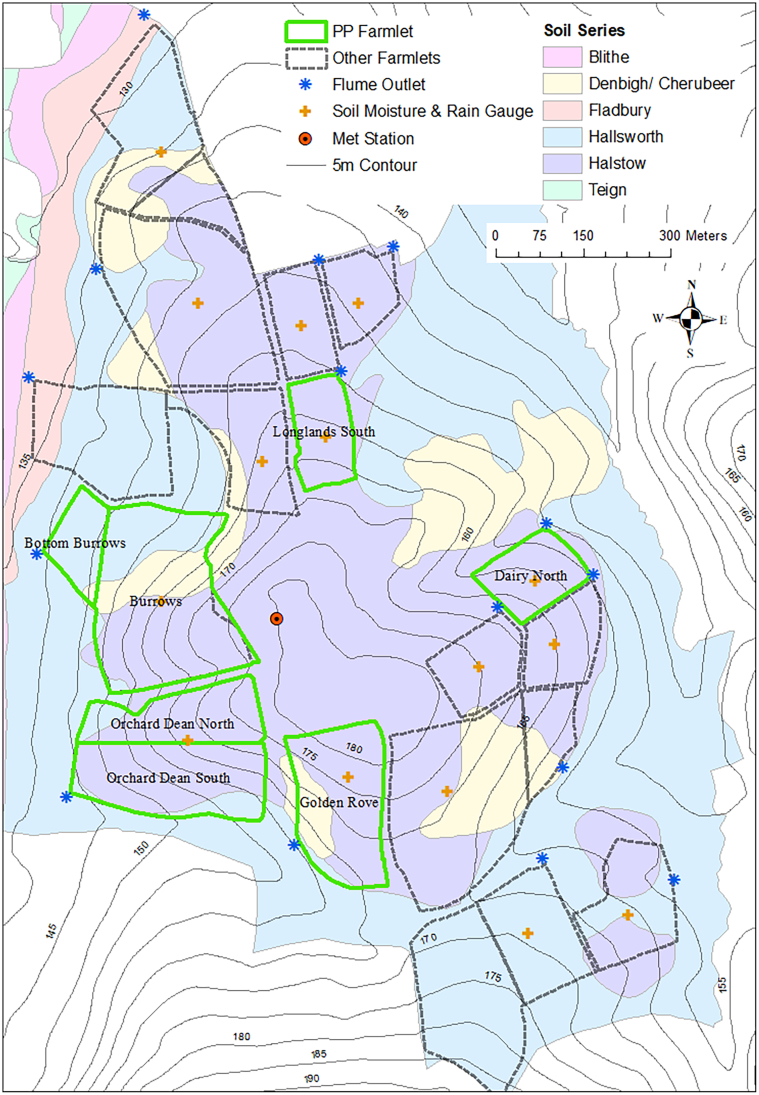
Map of the North Wyke Farm Platform (NWFP), showing the permanent pasture (PP) farmlet, sub-catchments, fields, soil class, topography and the locations of flume outlets where water and nutrient fluxes are measured. The soil moisture and rain gauge in Top Borrows is situated within the North Wyke Met station.

Simulated animal performance was validated with data collected from the NWFP from 2011 to 2018 (50°46′10″N, 3°54′05″W and 120–180 m a.s.l.). North Wyke has a temperate climate with average annual precipitation of 1030 mm and mean daily minimum and maximum temperatures of 7.0 and 13.6 °C, respectively, from 1989 to 2018. The site overlays clay shales and the predominant soil type is a Stagni-vertic Cambisol under the FAO classification (Harrod and Hogan, 2008), which comprises a slightly stony clay-loam topsoil, overlying a mottled stony clay derived from the carboniferous culm measure. The platform is a 63 ha systems-based experimental facility divided into three 21 ha farmlets (small farms) with five hydrologically isolated sub-catchments in each. Over the simulation period, the farmlet treatments (pasture-type) were one of permanent pasture (PP) predominantly perennial ryegrass (*Lolium perenne* L.), monoculture reseed with high sugar perennial ryegrass (*L. perenne* cv Aber Magic) and a reseed mixture of high sugar perennial ryegrass and white clover (*Trifolium pratense* L.). From April 2011 to March 2013, the baseline period, all three farmlets were as one (PP) with no separate treatments in operation. From April 2013 to September 2015, the two reseed farmlets transitioned into a post-baseline phase with the third continuing as PP. Thus, some sub-catchments entered a post-baseline phase much earlier (say in 2013) than others (say in 2015). Given this and to furnish a long time series of consistent / coherent data for a robust calibration, validation and interpretation of the SPACSYS simulations, only outputs from the PP farmlet (Fig. 3) were reported in this study. The size of each the five sub-catchments and the seven fields / paddocks for the PP farmlet together with management activities are shown in Table 1.

**Table 1:** Paddock size (ha) for various management activities on the permanent pasture (PP) farmlet.

Field name	Hydrological area	Fenced area	Area for cutting	Area covered for chemical fertiliser	area covered for manure application
Bottom Burrows^a^	1.34 of 8.08	1.26	1.20	1.23	0.99
Burrows^a^	6.73 of 8.08	6.49	6.38	6.43	5.74
Golden Rove	3.95	3.86	3.77	3.78	3.28
Dairy North	1.87	1.78	1.73	1.74	1.39
Longlands South	1.81	1.75	1.69	1.69	1.42
Orchard Dean^b^	6.73	6.47	6.39	6.38	5.58
South	4.05	3.92	3.84	3.85	3.34
North	2.68	2.55	2.47	2.51	2.14

a Together forms a single sub-catchment where a further field.

b Sub-catchment split into two fields from mid-August 2015: Orchard Dean South and Orchard Dean North. We reported the results from these split fields as a single unit.

For the study period from 2011 to 2018, each farmlet was grazed by 30 finishing beef cattle and typically 75 ewes with their lambs (typically 135 assuming a lambing rate of 1.8). Cattle were introduced to the farm platform after weaning, at 6 months of age, and were initially housed over the winter period (typically from October to March) and fed silage harvested from their respective farmlet, and then grazed on their respective farmlet at turnout until removed for slaughter on achieving a target weight of 555 kg (heifers) / 620 kg (steers) and fat class (4 L). Ewes typically grazed into the winter season (late November to early January) and were then housed and fed off the platform prior to lambing, which normally occurs between mid-March and early April; they were subsequently returned to the platform (typically March/April) with their lambs, which were finished at a target weight of 43 kg and fat class (3 L). All animal movements were recorded using unique identifier tags. Prior to 2017, a Hereford x Friesian herd provided predominantly Continental x calves, with heifers first calved to a Hereford bull. The breeding herd was subsequently transitioned to Stabilisers™. In total, seven breeds dominate: Charolais cross (CHX), Hereford (HEX), Limousin (LIMX), Stabiliser cross (STX), Stabilisers (ST), Simmental cross (SMX) and Belgian Blue cross (BRBX). Lamb were predominantly progeny of Suffolk x Mule (SUFMU) ewes crossed with Charolais (CHA) or Lleyn (LLE) rams.

In total, data for over 200 finishing beef cattle and 900 lambs were used in this study for the period 2011 to 2018. This resulted in 1383 periodic liveweight beef cattle measurements (across the above seven cattle breed combinations) and 3997 periodic liveweight lamb measurements (across the above two sheep breed combinations) for use in model performance assessment.

### 2.4 Simulation configurations

The SPACSYS model has previously been validated using the NWFP data in terms of water fluxes, N_2_O emissions, grass biomass accumulation and soil C and N budgets (Carswell et al., 2019b; Li et al., 2017; Liu et al., 2018; Wu et al., 2016). Hence, all the initialised states and parameters for soil water redistribution, heat transformation, grass growth and soil C and N cycling were adopted from previous studies. No further validation on these variables was made for this study. Information on agronomic management, animal movement and liveweight was freely accessed and downloaded from the NWFP data portal (http://resources.rothamsted.ac.uk/farmplatform). In most study years, liveweight was measured once every two weeks, while in the latter years this was reduced to once every four weeks.

The growth rate of each animal was simulated from its first grazing day in a field of the PP farmlet to its last day in the PP farmlet. The record of the birth date and liveweight at the beginning of grazing for each animal was used as model input for the initial weight and age of the animal. For each animal, dates of movements between fields were used to determine the grazing period within a given field. It was assumed that feed supply during the off-paddock periods was sufficient to meet the animal requirements. Weaning date for lambs each year is set at the end of June. For ewes, if there was no initial weight recorded, a default value of 70 kg was applied.

Annual NH_3_, CH_4_ and CO_2_ from both animals and soils and N_2_O from soils were simulated and N cycling in each field was analysed. A hydrological year from April to March was used to calculate annual values.

### 2.5 Statistical analysis

To assess the performance of the finishing beef cattle and lamb liveweight simulations, six accuracy indices were found (the mean error (MErr), the percentage bias (PBIAS), the mean absolute error (MAE), the normalized root mean square error (NRMSE), the Nash-Sutcliffe efficiency (NSE), and the Kling-Gupta efficiency (KGE)), which can be respectively defined as:

(43) $$\text{MErr}=\frac{1}{N}\sum_{i=1}^{N}{\hat{z}}_{i}-z_{i}$$

(44) $$\text{PBIAS}=100\frac{\sum_{i=1}^{N}\left({\hat{z}}_{i}-z_{i}\right)}{\sum_{i=1}^{N}z_{i}}$$

(45) $$\mathrm{MAE}=\frac{1}{N}\sum_{i=1}^{N}|{\hat{z}}_{i}-z_{i}|$$

(46) $$\text{NRMSE}=100\frac{\sqrt{\frac{1}{N}\sum_{i=1}^{N}{\left({\hat{z}}_{i}-z_{i}\right)}^{2}}}{z_{\max}-z_{\min}}$$

(47) $$\mathrm{NSE}=1-\frac{\sum_{i=1}^{N}{\left({\hat{z}}_{i}-z_{i}\right)}^{2}}{\sum_{i=1}^{N}{\left(z_{i}-{\bar{z}}_{i}\right)}^{2}}$$

(48) $$\mathrm{KGE}=1-\sqrt{{\left(r-1\right)}^{2}+{\left(\frac{\sigma_{\hat{z}}}{\sigma_{z}}-1\right)}^{2}+{\left(\frac{\bar{\hat{z_{i}}}}{{\bar{z}}_{i}}-1\right)}^{2}}$$

Where *N* is the total paired number, ${\hat{z}}_{i}$ are simulated liveweight values, *z*_*i*_ are measured liveweight values,$\;{\bar{\hat{z}}}_{i}$ is the mean of the simulated values, ${\bar{z}}_{i}$ is the mean of the measured values, *z*_*max*_ and *z*_*min*_ are the maximum and minimum values among the measured data, respectively. Further, *r* is the Pearson product-moment correlation coefficient (between simulated and measured) and $\sigma_{\hat{z}}$ and *σ*_*z*_ are the standard deviations for the simulated and measured data, respectively.

The ideal value of the four error-based indices (MErr, PBIAS, MAE and NRMSE) is zero such that the closer to zero, the more accurate the model simulation. Negative MErr and PBIAS values indicate a tendency to under-prediction, while positive values indicate a tendency to over-prediction of liveweight by the model. NSE takes values from −∞ to 1, where unity corresponds to an exact match between simulated and measured data, zero indicates that the simulations are as accurate as the mean of the measured values and a negative value indicates that the simple arithmetic mean of the measured is a better predictor than the model. KGE incorporates the correlation coefficient *r*, the ratio between the means of the simulated and of the measured data and the variability ratio. As with NSE, KGE takes values from −∞ to 1. MErr and PBIAS provide complementary error indices for over- and under-prediction, MAE and NRMSE provide complementary error indices for prediction accuracy, while NSE and KGE provide complementary indices for levels of agreement between simulated and measured data. Performances indices are calculated using the ‘hydroGOF’ R package. A seventh model performance index is also reported with the usual *R*^2^ value (the coefficient of determination) for a regression fit to the simulated and measured data. Performance indices are found across different animal ages, breeds and grazing years.

Using simulated outputs only, one-way ANOVAs were used to test differences in liveweight, growth rate, CH_4_ and CO_2_ emissions between cattle and sheep breeds. Note for CH_4_ and CO_2_ emissions, no model validation data exist.

## 3 Results

### 3.1 Model performance assessment with measured data

**Fig. 4:**
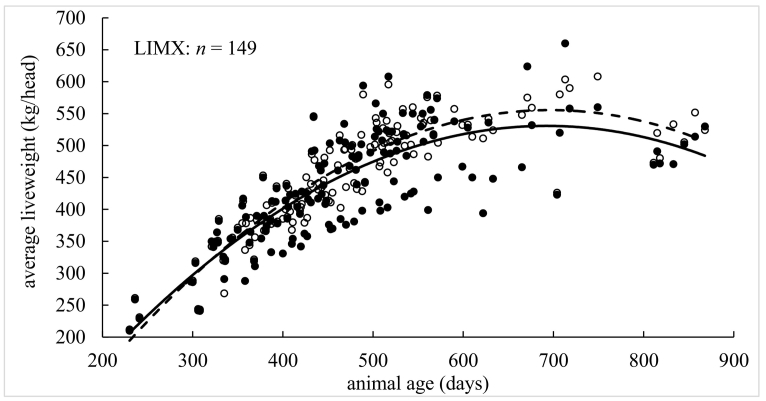

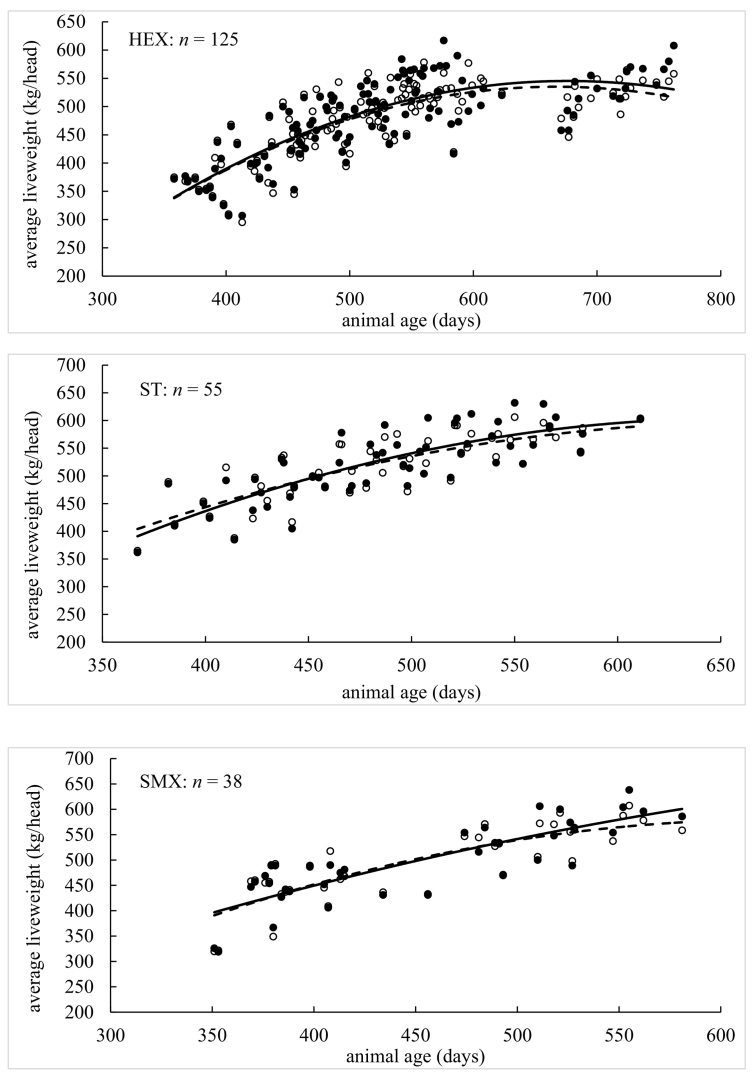

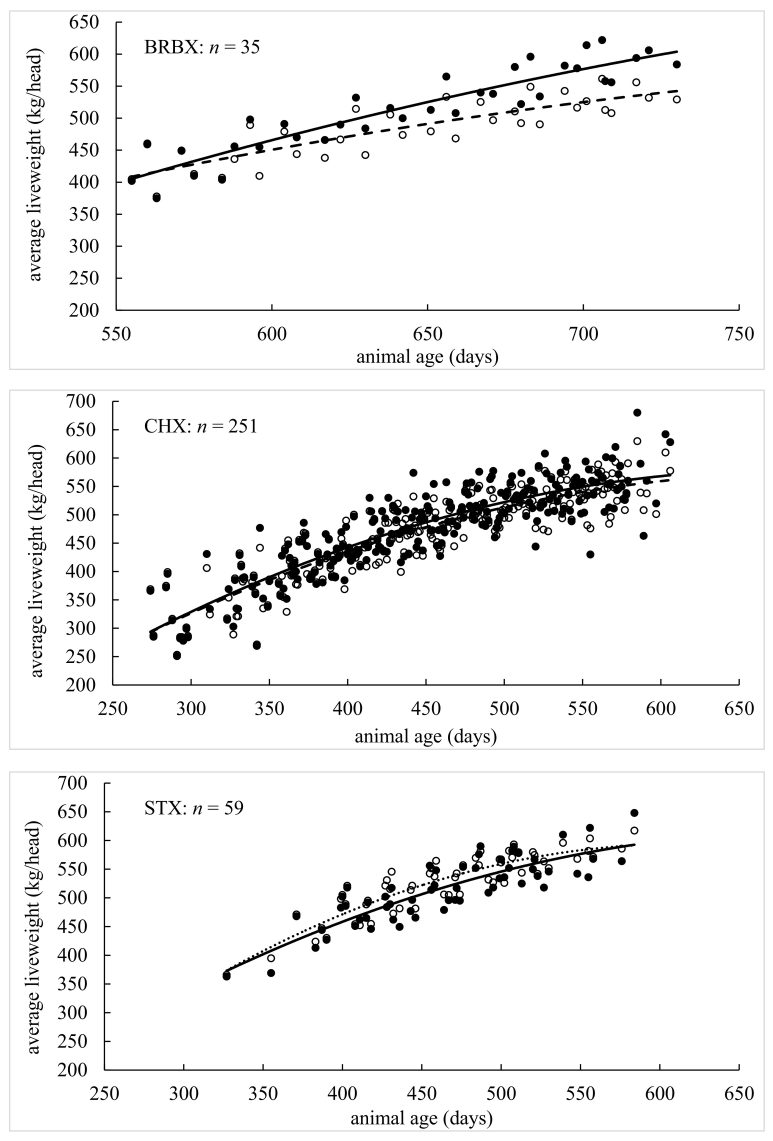

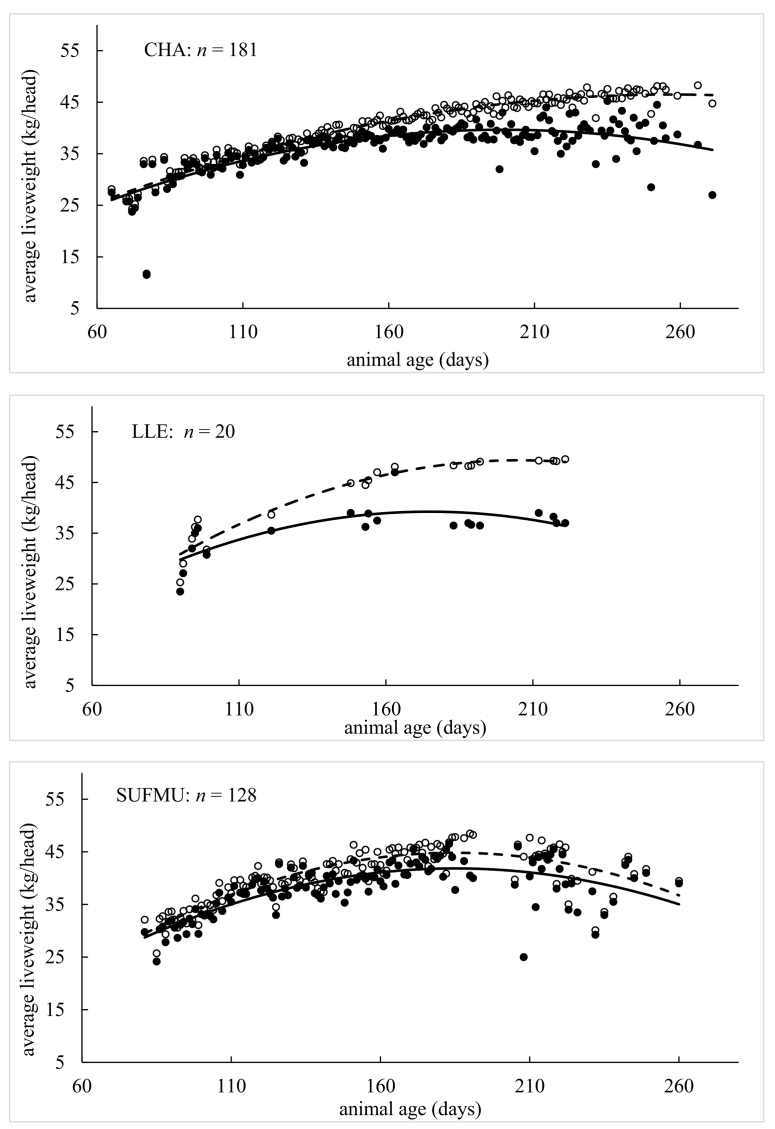
Comparison of measured and simulated animal liveweight by age for given breeds (BRBX, CHX, HEX, LIMX, SMX, ST and STX for cattle; CHA, LLE and SUFMU for lambs), where *n* is number of observations. Solid circles are measured average liveweight and open circles are simulated average liveweight. Solid and dotted lines are fitted polynomial functions for measured and simulated average liveweight against age, respectively.

Model performance indices *per individual* liveweight measurement are presented in Table 2, Table 3. Performance indices (MErr, PBIAS, MAE, NRMSE, NSE, KGE and *R*^2^) were found conditional to age, breed and grazing year. Graphical depictions of model performance according to breed are presented in Fig. 4, where animal age was plotted against *average* liveweight for simulated and measured data. In each case, the plots for simulated and measured data were fitted with polynomial functions so that simulated (on average) growth curves could be visually assessed against measured (on average) growth curves. The first model assessment (Table 2, Table 3) is more detailed as it is conducted on each *individual* liveweight measurement, while the second assessment (Fig. 4) is broader as it is conducted on *average* liveweights.

**Table 2:** Model performance on beef cattle by age (days), breed and grazing year.

Age	All	<300	300–360	360–420	420–480	480–540	540–600	600–660	660–720	720–780	780+
Sample size	1383	18	55	236	360	398	206	34	43	22	11
MErr	−1.32	2.35	−2.54	0.23	−1.71	−4.20	−3.79	15.28	−4.33	22.30	41.67
PBIAS %	−0.30	0.80	−0.70	0.10	−0.40	−0.80	−0.70	3.00	−0.80	4.10	8.30
MAE	21.63	2.37	10.41	11.65	18.41	22.34	29.42	42.15	42.68	56.12	42.70
NRMSE %	38.50	4.70	31.80	32.60	41.50	49.20	60.20	88.80	89.00	129.10	191.9
NSE	0.85	1.00	0.90	0.89	0.83	0.76	0.64	0.19	0.19	−0.75	−3.05
KGE	0.90	0.99	0.92	0.92	0.90	0.81	0.69	0.46	0.39	−0.35	0.19
*R*^*2*^	0.85	1.00	0.90	0.89	0.83	0.76	0.64	0.29	0.23	0.07	0.30
Breed		LIMX	HEX	ST	SMX	BRBX		CHX			STX
Sample size		199	168	64	40	57		771			84
Animal No.		27	32	10	7	8		130			14
MErr		13.23	−7.54	−4.53	−3.48	−3.56		−4.80			13.58
PBIAS %		3.00	−1.60	−0.90	−0.70	−0.70		−1.00			2.60
MAE		22.24	23.54	13.28	10.78	42.74		21.11			18.36
NRMSE %		37.20	45.60	29.50	18.60	52.00		38.80			40.30
NSE		0.86	0.79	0.91	0.96	0.72		0.85			0.84
KGE		0.93	0.86	0.87	0.95	0.69		0.91			0.91
*R*^*2*^		0.89	0.80	0.92	0.97	0.75		0.85			0.89
Grazing year		2011	2012	2013	2014	2015		2016		2017	2018
Sample size		141	134	115	171	257		204		171	190
MErr		−4.16	14.35	5.52	−0.84	−20.77		5.23		0.87	2.47
PBIAS %		−0.90	3.20	1.20	−0.20	−4.40		1.00		0.20	0.50
MAE		11.94	25.79	30.43	20.74	34.47		16.24		13.02	17.54
NRMSE %		18.60	38.90	60.90	43.50	50.30		33.30		23.20	36.20
NSE		0.97	0.85	0.63	0.81	0.75		0.89		0.95	0.87
KGE		0.97	0.87	0.81	0.81	0.84		0.88		0.94	0.84
*R*^*2*^		0.97	0.88	0.66	0.81	0.80		0.90		0.95	0.88

**Table 3:** Model performance on lamb by age (days), breed and grazing year.

Age	All	<120	120–140	140–160	160–180	180–200	200–220	220–240	260+
Sample size	3674	1491	591	600	473	250	176	60	33
MErr	2.29	1.01	1.62	2.67	3.45	4.47	5.70	5.74	7.57
PBIAS %	6.3	3.1	4.4	7.0	8.8	11.3	14.5	14.9	19.7
MAE	2.79	1.40	2.49	3.39	3.93	4.80	5.75	5.78	7.57
NRMSE %	70.4	34.3	64.6	95.3	110.3	150.3	151.4	149.6	198
NSE	0.50	0.88	0.58	0.09	−0.22	−1.27	−1.30	−1.27	−3.04
KGE	0.83	0.91	0.74	0.61	0.52	0.32	0.22	0.57	0.13
*R*^*2*^	0.72	0.92	0.70	0.47	0.41	0.16	0.16	0.36	0.08
Breed		SUFMU			CHA			LLE	
Sample size		671			2921			34	
Animal No.		282			575			35	
MErr		2.38			2.27			6.82	
PBIAS %		6.4			6.3			19.3	
MAE		2.57			2.82			6.82	
NRMSE %		59.6			72.8			154.2	
NSE		0.64			0.47			−1.45	
KGE		0.88			0.82			0.43	
*R*^*2*^		0.80			0.70			0.67	
Grazing year		2011	2012	2013	2014	2015	2016	2017	2018
Sample size		341	356	203	286	317	730	731	710
MErr		1.04	2.10	2.73	0.45	0.81	3.42	1.54	3.86
PBIAS %		2.7	5.8	7.3	1.2	2.1	9.7	4.3	11.2
MAE		1.96	2.60	3.45	1.70	2.12	3.50	2.00	3.93
NRMSE %		49.1	57.2	83.0	43.7	70.5	92.9	50.8	93.5
NSE		0.76	0.67	0.31	0.81	0.50	0.14	0.74	0.12
KGE		0.87	0.86	0.74	0.87	0.77	0.79	0.90	0.75
*R*^*2*^		0.80	0.78	0.64	0.82	0.60	0.73	0.83	0.73

For beef cattle, all accuracy indices (Table 2) suggest model performance moves from a high to a low level of accuracy as animals age. There was no consistent over- or under-prediction given MErr and PBIAS could be both positive and negative. Model accuracy was poor for animals that were aged around 600 days and over (e.g., with NSE dropping to 0.19 and *R*^2^ dropping to 0.29 for the 600- to 660-day class). Although this threshold coincided with a sharp decrease in observations as animals reached their target weight ready for slaughter. For cattle breed, all accuracy indices suggest little difference in model performance, where prediction accuracy was commonly strong, and where under-prediction was more likely than over-prediction (as MErr and PBIAS tended to be negative). Strongest levels of model accuracy were found for the SMX breed (with NSE, KGE and *R*^2^ values all close to unity), while weakest levels of accuracy were found for BRBX cattle. Both SMX and BRBX breeds were relatively small in number, where the predominant breeds (LIMX, HEX and CHX) were all predicted with strong levels of accuracy. For cattle by grazing year, all accuracy indices indicate strong levels of model performance with say, *R*^2^ values >0.80 and NSE values >0.75, but with the notable exception of the 2013 grazing year where *R*^2^ = 0.66 and NSE = 0.63. In summary, overall model accuracy for simulating cattle liveweight (regardless of age, breed or grazing year) was strong with NSE = 0.85, KGE = 0.90 and *R*^2^ = 0.85 (i.e. all three indices were close to unity).

Similar to cattle, all indices for lambs (Table 3) suggest model performance moves from high to low levels of accuracy as animals age. However, for lambs, prediction accuracy became weak at a relatively early age, with NSE dropping to 0.09 and *R*^2^ dropping to 0.47 for animals in the 140- to 160-day class (and this performance became weaker still for all remaining ages). There was also a consistent over-prediction of liveweight for each age class given that MErr and PBIAS were always positive. For lamb breed, the accuracy indices were more diverse and harder to interpret, where moderate to strong levels of accuracy were found for the dominant SUFMU and CHA breeds (with *R*^*2*^ and NSE values of 0.80 and 0.64, and 0.70 and 0.47, respectively), while moderate to weak levels of accuracy were found for the LLE breed (i.e. an *R*^*2*^ = 0.67 coupled with a poor NSE = −1.45). Again, there was a consistent over-prediction of liveweight given that MErr and PBIAS were always positive. For lambs by grazing year, most accuracy indices suggest moderate to strong levels of model performance across all eight years with, *R*^2^ values >0.60 and KGE values >0.74. However, the NSE index only suggested moderate to strong levels of model performance for some years (say 2011, 2012, 2014, 2017 with NSE > 0.51) and not others (say 2013, 2015, 2016, 2018 with NSE < 0.51). In summary, overall model accuracy for simulating a lambs liveweight (regardless of age, breed or grazing year) was moderate to strong with NSE = 0.50, KGE = 0.83 and *R*^2^ = 0.72 (i.e. one out of three indices were close to unity).

For the graphical descriptions of model performance, where *average* liveweights are assessed against age (Fig. 4), liveweight for all seven cattle breeds was simulated with strong levels of accuracy (as the fitted polynomials were highly similar). However, such levels of accuracy could weaken as cattle get older, confirming that found above. For the BRBX breed, the model tended to under-predict liveweight (as the fitted polynomial to the simulated data mostly lies below that for the fitted polynomial to the measured data), while conversely the model tended to over-predict liveweight for the LIMX breed. Such clear under- or over-prediction was not present for the remaining five breeds. Liveweights for the lambs tended to be over-predicted for all three breeds, where this over-prediction was stronger as the lambs aged for both CHA and LLE breeds. Overall, model simulation accuracy for a lamb's liveweight was weaker than that found for a cow's liveweight.

### 3.2 Simulated performance for different breed combinations

Simulated liveweight gain and gaseous emissions rates during the grazing period from individual beef and sheep (lamb) breed combinations are shown in Table 4. For cattle, STX emitted the least CH_4_ per head compared with other cattle breed combinations, while SMX had the highest emission, although when expressed on a per LWG basis there were no significant differences. There was no significant difference in CO_2_ respiration among the cattle breed combinations. There was a significant difference in the growth rate between sheep breed combinations, with LLE at the greatest rate and CHA at the least. Across the sheep breed combinations, CHA showed the lowest emissions of both CH_4_ and CO_2_.

**Table 4:** Simulated average daily liveweight gain (kg d^−1^) and methane and carbon dioxide emissions (g head^−1^ d^−1^) during the grazing period for beef and sheep (lamb) breed combinations (different letters in a column either for cattle or sheep indicate a significant difference among breed combination, *p* < 0.05).

Breed	Animal No.	Average liveweight	Growth rate (LWG)	CH_4_		CO_2_
		kg	kg head^−1^ d^−1^	g head^−1^ d^−1^	g LWG^−1^	g C head^−1^ d^−1^
Cattle						
CHX	130	475	0.80^a^	265^ab^	331.3^a^	2045^ab^
HEX	32	468	0.83^a^	268^ab^	322.9^a^	2043^ab^
LIMX	27	448	0.80^a^	267^ab^	333.8^a^	1938^a^
STX	14	533	0.72^b^	242^b^	336.1^a^	2184^b^
ST	10	525	0.70^b^	245^b^	350.0^a^	2133^ab^
BRBX	8	484	0.75^ab^	259^ab^	345.3^a^	2028^ab^
SMX	7	502	0.78^ab^	289^a^	370.5^a^	2125^ab^

Sheep						
SUFMU	282	41	0.23^a^	26.9^a^	126.1^a^	294^a^
CHA	575	40	0.17^b^	22.3^b^	138.9^a^	273^b^
LLE	35	41	0.29^c^	30.7^c^	110.0^a^	313^c^

### 3.3 Simulated gaseous emissions

Averaged annual emissions of GHGs and ammonia from different sources over the simulation period are shown in Table 5. There was less variation in N_2_O and CO_2_ emissions from plants and soils between fields than for NH_3_, CH_4_ and animal-derived CO_2_ emissions, which relate to animal type (cattle or sheep), stocking density and duration in each field. For example, annual stocking density is 340 head·d ha^−1^ for cattle and 412 head·d ha^−1^ for sheep in Burrows but in Golden Rove annual stocking density is 224 head·d ha^−1^ for cattle and 1500 head·d ha^−1^ for sheep.

**Table 5:** Average annual (April – March) gaseous emissions (kg ha^−1^) from soils (heterotrophic respiration), plants (autotrophic and above-ground dark respiration) and animals when they grazed from 2011 to 2018 for each field of the PP farmlet.

Field	N_2_O - N	NH_3_ - N	CH_4_	CO_2_		
				plant	soil	animal
Bottom Burrows	2.7	8.6	98	7520	5691	979
Burrows	3.0	11.5	105	6914	6277	854
Dairy North	2.3	9.9	171	6657	4963	1808
Golden Rove	2.8	6.9	101	6233	5289	892
Longlands South	2.7	8.4	189	6709	5302	1716
Orchard Dean^a^	3.1	9.9	91	6344	5522	790
Orchard Dean North^b^	3.0	7.1	103	5112	5755	958
Orchard Dean South^b^	2.7	9.3	124	6955	6099	941

a Before the field was split.

b After the Orchard Dean field was split.

### 3.4 Nitrogen cycling

Averaged annual N inputs to and outputs from the individual fields over the simulation period are shown in Table 6. Total N input ranged from 190 to 260 kg ha^−1^. Between 37 and 60% of the N added to the fields was removed through harvested biomass (silage) or animal intake, and 15–26% of N was lost through surface runoff or lateral drainage. Annual averaged gaseous losses of N_2_O and NH_3_ over the simulated period were 2.77 ± 0.24 and 8.95 ± 1.54 kg N ha^−1^, respectively.

**Table 6:** Average annual (April – March) N inputs and outputs (kg N ha^−1^) from each field of the PP farmlet from 2011 to 2018.

Field	Input				Output				
	Deposition	Fertiliser	Manure	Excreta	Cut	Animal intake	Volatilisation	Leach & runoff	Denitrification
Bottom Burrows	20.1	144.6	34.2	29.0	59.4	36.9	8.6	37.8	34.2
Burrows	20.0	164.3	63.8	31.9	63.8	41.8	11.5	46.1	39.3
Dairy North	20.0	142.3	0.0	49.4	14.9	54.9	9.9	42.5	30.8
Golden Rove	20.0	171.6	12.8	30.3	66.9	38.3	6.9	41.8	35.2
Longlands South	20.0	155.5	0.0	49.2	35.8	56.3	8.4	44.5	32.5
Orchard Dean^a^	20.5	158.8	60.1	29.1	51.5	38.1	9.9	49.9	43.2
Orchard Dean North^b^	19.3	155.8	50.4	34.5	91.5	44.8	7.1	34.0	41.7
Orchard Dean South^b^	19.2	146.6	48.5	31.5	45.3	41.9	9.3	39.1	40.3

a Before the field was split.

b After the Orchard Dean field was split.

## 4 Discussion

### 4.1 Model performance of beef finishing cattle and sheep growth

For both beef cattle and sheep, individual animals differ in their growth rate and their health status naturally within any livestock enterprise. Growth rates will similarly vary between breeds and the change in meteorological conditions during each grazing season. Given this, when objectively assessing model performance on simulating liveweight of cattle and sheep, for different age ranges, breed combinations and grazing seasons (Table 2, Table 3 and Fig. 4), the extended SPACSYS model could accurately simulate the dynamics of animal liveweight within the natural variations expected. Relatively, liveweight simulations for cattle were shown to be more accurate than those for sheep, where in both instances, simulation accuracy weakened as animals aged, which possibly is the result of the estimation of the potential growth rate. Further, levels of accuracy differed more across sheep breeds than it did across cattle breeds. Grazing year could also influence simulation accuracy, although reasons for this are not entirely clear.

The extended SPACSYS model is capable of simulating not only animal growth but also other elements of livestock (either beef finishing cattle or sheep) production at a systems level. Therefore, the model has the potential to investigate the responses of the system on and consequences of a range of agronomic management and grazing strategies – i.e., not only those as analysed across the farmlet (small farm) of this research with its specific (single) management and (single) grazing approach.

### 4.2 Gaseous emissions from cattle and sheep

The simulated averaged CH_4_ emission rate was between 242 and 289 g head^−1^ d^−1^ for beef cattle and between 22 and 31 g head^−1^ d^−1^ for sheep aged between three and seven months (Table 4). There are few measurement datasets available for UK grazing systems, but the simulated data are within the expected range according to those datasets that have been published and, more broadly, with the default values provided by the IPCC Guidelines for national GHG inventories (IPCC, 2019). Meo-Filho et al. (2021) reported average emission rates of 183–213 g head^−1^ d^−1^ for growing beef cattle grazing the same PP farmlet of the NWFP during late summer of 2019, measured using the SF_6_ tracer gas technique (Berndt et al., 2014). Fraser et al. (2014), also using the SF_6_ tracer gas technique, measured emissions from upland and lowland grazing beef cattle and reported emissions in the range 173–217 g head^−1^ d^−1^. For sheep, using an emission chamber methodology and a cut and carry system for feeding fresh herbage, Moorby et al. (2015) measured emissions from mature ewes fed permanent pasture of 11–15 g head^−1^ d^−1^_,_ and Fraser et al. (2015) reported emission rates in the range 12–17 g head^−1^ d^−1^ for growing lambs. More generally, default emission rates provided by the IPCC (2019) equate to 142 and 25 g head^−1^ d^−1^ for finishing beef cattle and productive sheep in Western Europe, respectively. While there were significant differences between breed combinations in the simulated emissions per head for both beef cattle and sheep (Table 4), the literature evidence is that breed is a far less important variable (generally non-significant) influencing CH_4_ emission than other factors such as diet characteristics and feed DMI (Duthie et al., 2017; Fraser et al., 2014; Fraser et al., 2015; Moorby et al., 2015). Any differences in emissions per head between breeds are generally accounted for through differences in body size, productivity or feed intake and, therefore, on an emission intensity basis (CH_4_ kg^−1^ LWG) breed is considered relatively unimportant.

There was less variation in respiration rate between different beef and sheep breed combinations (Table 4) suggesting that breed plays only a minor role and that body size is the major determinant of the respiration rate (data not shown). Although a direct comparison with measurement data is lacking, relative errors of less than 10% between the simulated and reported values for animals of the same size (Chaves et al., 2006; Gunter and Beck, 2018) support the model output. There have been few measurements, reported to date on CO_2_ emissions from sheep. In an early study, Whitelaw et al. (1972) reported that an average of 232 g CO_2_-C head^−1^ d^−1^ was produced by sheep weighing 56–78 kg at 12 °C ambient temperature, which is slightly lower than we estimated for an animal with an average body size of 40 kg.

### 4.3 Nitrogen cycling

Averaged annual N input to the individual farmlet fields ranged from 190 to 260 kg ha^−1^, which mainly reflected variations in stocking density and duration across fields (Table 6). The estimated output components in N balance are within the range of the reported values. For example, an annual average loss rate through surface runoff or lateral drainage of 42 (±5) kg N ha^−1^ over the farmlet, which was close to the estimate from the NWFP in a previous study (Shepherd et al., 2019).

An annual average of 2.77 (±0.24) kg N ha^−1^ as N_2_O over the simulated period was emitted to the atmosphere. Although as a proportion of the total input this is small and agronomically of little consequence, it is of environmental significance because of the high global warming potential of N_2_O. Sources for this emission include the atmospheric N deposition, the applied fertiliser N and farm-yard manure (FYM) N as well as the in-field recycled N being deposited as dung and urine by the animals (making the N content of the grazed herbage available to the soil microbial processes of nitrification and denitrification) and N from senescent above- and below-ground plant material. The simulated N_2_O emission was equivalent to 1.14 ± 0.05% of the N input for these sources. This estimate is a composite of the various N_2_O sources and therefore difficult to compare with emission factors reported elsewhere for individual N sources. It is in the range of 0.1–1.8% given as the default emission factor (EF_1_) by IPCC (2019) for fertiliser and FYM N additions to the soil, and the range for the default IPCC emission factor (EF_3_) of 0–1.4% for cattle excreta returns during grazing (IPCC, 2019). It is also of a similar order of magnitude to empirical data from recent UK studies. Cowan et al. (2020) reported an average value of 1.33% for synthetic N fertiliser (ammonium nitrate) based on 202 observations for grassland soils in the UK and Ireland. Thorman et al. (2020) reported an average emission for FYM applied to grassland of 0.37%, based on three experimental sites, with a value of 0.13% specific to the North Wyke site. Chadwick et al. (2018) analysed available UK data for N_2_O emissions from cattle dung and urine returns to soil, developing average emission factors of 0.69 and 0.19% for urine and dung, respectively, based on five sites and applications at three times of the year across the grazing season. There are large uncertainties in these estimated emission factors for agricultural soils because of many influencing environmental and management factors (Cowan et al., 2020).

Agriculture is the major source of NH_3_ emissions to the atmosphere, primarily deriving from livestock excreta, including manure and urea / NH_3_-based fertiliser applications (Behera et al., 2013). In SPACSYS, NH_3_ volatilisation from chemical fertilisers is not yet considered. Ammonium nitrate was applied in this study, which is associated with much lower NH_3_ emissions than other fertiliser types, e.g. urea (Forrestal et al., 2016), typically of less than 5% of the applied fertiliser N (e.g. Misselbrook et al., 2004). We simulated NH_3_ volatilisation from applied FYM and excretal grazing returns at an average annual value of 8.95 ± 1.54 kg N ha^−1^, equivalent to 12.9% of the FYM and excreta N. Ammonia emissions from applied FYM can be low, as the ammonium-N content of the FYM is typically low (Chambers, 2003), particularly for FYM that has previously been stored for some months, because of volatilisation losses and immobilisation processes during storage. Nicholson et al. (2017) quoted a mean emission for livestock FYM based on UK experiments of 4.5% of the total N applied while Misselbrook et al. (2005) reported a loss of 69% of the available N at spreading, which equated to approximately 8% of the total N applied. Emissions from excretal returns at grazing derive primarily from the urine (Laubach et al., 2013) and previous experiments in the UK and Netherlands give emissions typically in the range 5–10% of urine N deposited (Bussink, 1994; Jarvis et al., 1989; Jarvis et al., 1991; Lockyer, 1984), although Laubach et al. (2013) reported somewhat higher values (c. 25%) from trials in New Zealand. As with N_2_O emissions, NH_3_ emissions can vary considerably according to application techniques, N forms, soil texture, soil wetness and weather conditions at the times of application to the field. However, the rate might be underestimated in the model and should be further investigated, including an implementation of the NH_3_ volatilisation process from chemical fertilisers.

On average, the study farmlet annually received 208 kg N ha^−1^ and took 150 kg N ha^−1^ from the system (Table 6), which resulted in a surplus of 58 kg N ha^−1^. The imbalanced N budget suggested that the N application rate could be reduced to a certain extent or the livestock density might be increased to graze more forage during the grazing season. However, average simulated annual N uptake is 264 kg N ha^−1^ (data not shown). Considering the contribution from soil N mineralisation, the N budget could be balanced. Although volatilisation from FYM application or animal excreta has been considered, the loss from chemical N fertiliser through the process has not been included. It was reported that NH_3_ emissions represented 7 and 21% of the total applied N for ammonium-nitrate and urea, respectively, on grassland in the UK (Carswell et al., 2019a). Not including this loss in the model adds uncertainty to the N cycle.

### 4.4 Future development

As shown in this study, the extended SPACSYS model can dynamically simulate animal and grass growth, nutrient cycling and water redistribution in a soil profile considering the effects of animal genotype, climate, feed quality and quantity on livestock production, GHG emissions, water use and quality, and nutrient budgets at a field scale. It is novel to link animal, plant, soil and atmosphere together into a whole system model to quantitatively investigate the dynamics of animal and grass production and nutrient fate, and their interactions under varied environmental conditions. Through this study, the configuration for a permanent pasture grazing system has been validated. All PP farmlet fields were reasonably homogenous and dominated (>60%) by perennial ryegrass, with a smaller biomass of creeping bent (*Agrostis stolonifera*), Yorkshire fog (*Holcus lanatus*) and marsh foxtail (*Alopecurus geniculatus*) also contributing to the sward; legumes, on the other hand, comprised <1% of the overall composition (Takahashi et al., 2018). As more diverse, multi-species swards with higher proportions of legumes and forbs in intensive grasslands are becoming more common in practice, the modelling of these more diverse botanical composition swards needs to be validated as a subject of future work. Such modelling could also include the dynamics of individual species in the swards and their impact on animal growth and nutrient flows. Furthermore, animal intake preference and deliberate selection for specific species within the sward should be considered as it affects both the intake parameters and the subsequent development and composition of the sward, although this is challenging to implement in a process-based model. To date, no components have been implemented to simulate the impacts of extreme events such as temperature and rainfall, systematic animal-mediated nutrient transfers, pests, weeds and plant and animal genetic characteristics - environment interactions (GxE) on an agricultural ecosystem, which is highly desirable (Bryant et al., 2011). Evidence suggests that current guidance (Agricultural and Food Research Council, 1993) on nutritional requirements needs to be updated, where the ongoing research project (https://www.cielivestock.co.uk/improve-beef-feed-guidelines/) may lead to revisions to the energy requirements of beef cattle.

## 5 Conclusions

In this study, the extended SPACSYS model was shown to accurately and dynamically model finishing beef cattle, lamb and grass growth, nutrient cycling and water redistribution in a soil profile considering the effects of genotype, climate, feed quality and quantity on livestock production, GHG emissions, water use and quality, and nutrient cycling in a permanent pasture grazing system consisting of seven fields. Averaged annual N input to the individual fields ranged from 190 to 260 kg ha^−1^, of which 37–60% removed from the fields in terms of biomass cut or animal intake, and 15–26% through surface runoff or lateral drainage and 1.14% emitted to the atmosphere as N_2_O. About 13% of the FYM and excreta N in the farmlet volatilised from the soil. There are significant differences in animal growth rate, CO_2_ and CH_4_ emissions between different sheep breeds. However, there are less differences between the cattle breeds. Although the extended model was validated with data specific to Southwest England and for a permanent pasture grazing system, the model has clear potential to explore more innovative practices to maintain / increase livestock production whilst reducing adverse environment impacts across different livestock breeds, climates and soil types.

## Declaration of Competing Interest

The authors declare that they have no known competing financial interests or personal relationships that could have appeared to influence the work reported in this paper.
